# Pheochromocytoma During Pregnancy: A Hidden Cause for Hypertension

**DOI:** 10.7759/cureus.61286

**Published:** 2024-05-29

**Authors:** Sidonie Monteiro, Raquel Rodrigues, Amélia Almeida, Maria José Monteiro

**Affiliations:** 1 Obstetrics and Gynecology, Unidade Local de Saúde (ULS) do Médio Ave, Vila Nova de Famalicão, PRT; 2 Obstetrics and Gynecology, Unidade Local de Saúde (ULS) de Braga, Braga, PRT

**Keywords:** paroxysmal symptoms, catecholamine secreting tumor, pre-eclampsia, gestational hypertension, high-risk pregnancy, pheochromocytoma

## Abstract

Pheochromocytoma, a rare but potentially serious condition, poses challenges in timely identification, especially during pregnancy due to misconceptions about pregnancy-related hypertension causes. However, paroxysmal symptoms heighten diagnostic suspicion. The diagnosis relies on biochemical confirmation of catecholamine hypersecretion followed by imaging for tumor localization. When diagnosed at or after 24 weeks, alpha-adrenoceptor blockers are recommended during pregnancy to manage catecholamine excess, delaying tumor removal until viability or post-delivery. The rarity of this condition during pregnancy, coupled with diagnostic and management challenges, underscores its importance for obstetric professionals in addressing hypertensive control, delivery timing, and surgical intervention.

## Introduction

Pheochromocytoma is a tumor of the adrenal medulla that secretes catecholamines, leading to hypertensive crises. This disease is an uncommon yet potentially severe medical condition, primarily due to its association with hypertensive crises and the inherent life risk. The primary challenge lies in promptly diagnosing this etiology of hypertension during pregnancy to enhance maternal-fetal outcomes [1]. Although extremely rare, the frequency of new cases is estimated to be 0.002% [2]. Before 1970, studies reported high maternal and fetal mortalities (48% and 54%, respectively) when the diagnosis was missed antenatally [3]. However, other studies indicated significantly lower mortalities (maternal loss less than 5% and fetal loss less than 15%) when the diagnosis was made antenatally [4]. The rise in the percentage of patients with antenatal diagnosis and timely treatment has significantly contributed to reducing mortality.

## Case presentation

A 36-year-old pregnant woman, gravida 2, para 1, was referred to an obstetrics consultation at 12 weeks of gestation due to a diagnosis of chronic hypertension during her first pregnancy. Her medical history included gestational diabetes, obesity, and previous pregnancy preeclampsia.

At her initial prenatal visit, she was under therapy with methyldopa at maximum dosing, but it was inadequate to control the blood pressure. Even after the addition of a calcium channel blocker (nifedipine), the blood pressure did not meet in target values. She had several documented visits to the emergency department before the current pregnancy due to uncontrolled hypertension, palpitations, and night sweats. Her medical records documented consistently elevated blood pressure. Upon inquiry, the patient attributed her symptoms to episodes of anxiety.

**Figure 1:**
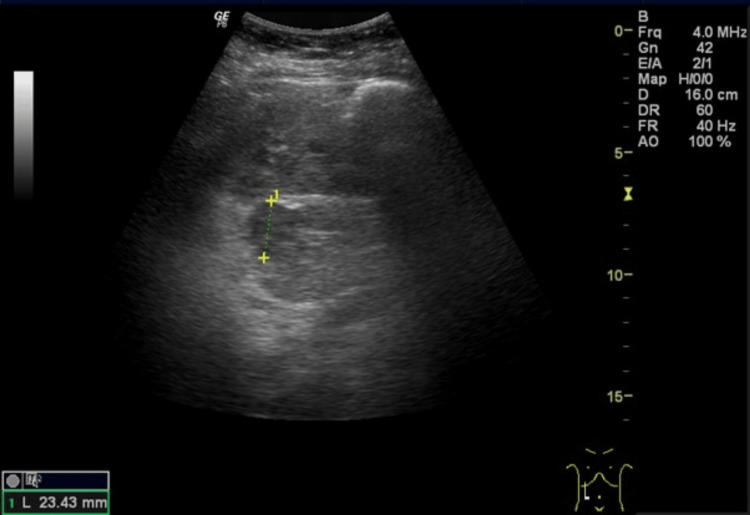
Renal ultrasound revealing the presence of a right pararenal mass measuring 23.43 mm.

Because of the challenging control, associated symptoms, and past history, a targeted biochemical and imaging study was conducted to investigate possible causes of secondary hypertension (HTA). Renal ultrasound revealed the presence of a right pararenal mass (Figure 1).

**Figure 2:**
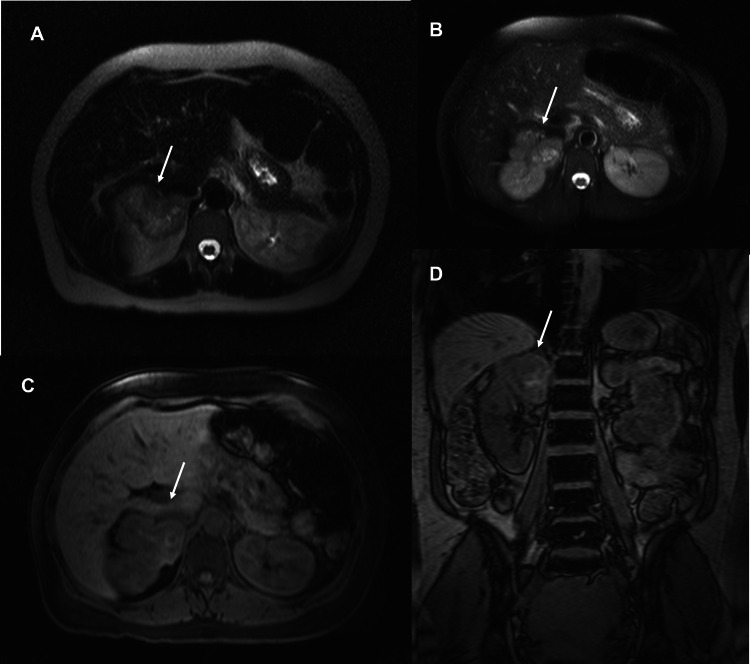
Abdominal MRI. An irregular and lobulated tumor lesion is observed in abdominal MRI images (A, B, C, D), poorly defined, adjacent to the upper pole of the right kidney: adrenal mass (arrow), from which it is inseparable, and it extends into the medial aspect of the kidney, reaching the renal hilum, along the path of the inferior vena cava, highly suggestive of pheochromocytoma.

Abdominal magnetic resonance imaging (MRI) confirmed the presence of an adrenal mass, highly suggestive of a locally advanced pheochromocytoma (Figure 2).

The assessment of 24-hour urinary fractionated metanephrines or plasma fractionated metanephrines confirmed the diagnosis of pheochromocytoma/paraganglioma (with a 10-fold elevation above the upper limit of normal values) at 24 weeks of gestation, which prompted the referral of the patient to a tertiary care center.

She initiated alpha-adrenergic blockade with phenoxybenzamine and maintained nifedipine to control blood pressure.

The case was discussed in a multidisciplinary meeting, and after involving the patient in the decision-making process, it was decided to maintain surveillance and postpone adrenalectomy until the postpartum period.

She was admitted to the hospital, where she was kept under close surveillance of maternal and fetal status.

At 28 weeks of gestation, the obstetric ultrasound evaluation revealed a fetus in the 12th percentile with umbilical artery (UA) index above 95th percentile. Despite being asymptomatic, the patient's persistent difficulty in controlling blood pressure prompted the request for an analytical study, which indicated a protein/creatinine ratio of 0.9 and an soluble fms-like tyrosine kinase 1 (sFlt-1)/placental growth factor (PlGF) ratio of 576, consistent with a diagnosis of superimposed preeclampsia.

At 29 weeks and one day of gestation, the obstetric ultrasound revealed a worsening of the Doppler evaluation with reversed diastolic flow in the UA and a middle cerebral artery index (IPACM) below the 5th percentile.

At this point, a decision was made to terminate the pregnancy. Magnesium sulfate was initiated as a protocol for fetal neuroprotection. The couple chose not to pursue fetal lung maturation after discussing the maternal risk and fetal benefit of corticosteroid therapy, due to the potential risk of hypertensive crisis associated with steroid administration. A cesarean section was performed under general anesthesia, and a male neonate weighing 1020 g with an Apgar score of 9/9/10 was delivered. The newborn was admitted to the neonatology intensive care unit and was discharged after 63 days, with good outcome and normal development at one year of age. The woman was discharged five days after delivery and maintained treatment with phenoxybenzamine and propranolol. The genetic study with next-generation sequencing was performed and excluded genetic causes for the pheochromocytoma.

**Figure 3:**
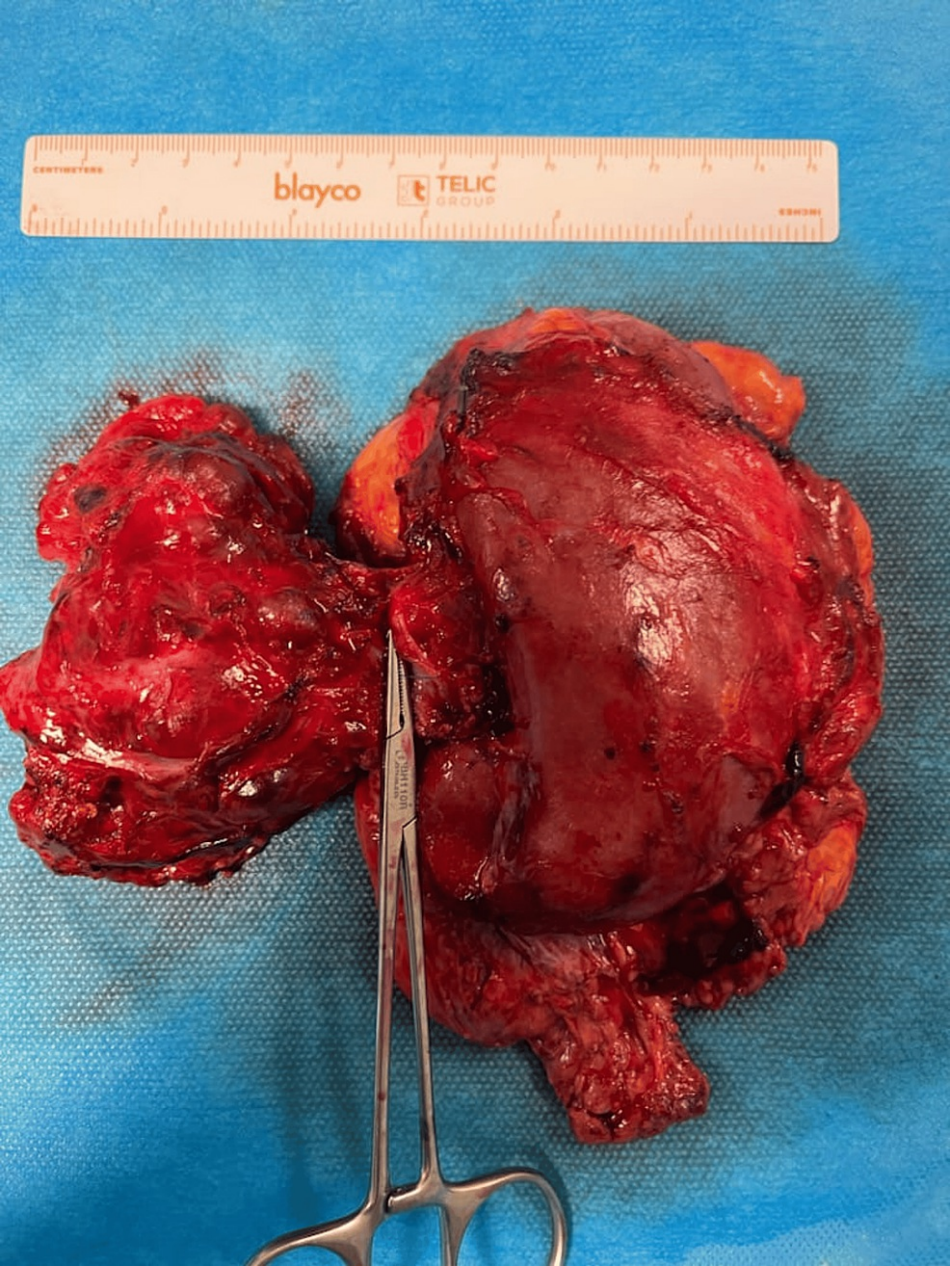
Excised surgical piece. The right adrenal tumor of 6.1 cm x 5.5 cm x 4.1 cm removed during surgery.

After adequate medical management for six months, she was submitted to laparoscopic right adrenalectomy and nephrectomy surgery due to absence of the cleavage plane with the renal vein. A right adrenal tumor of 6.1 cm x 5.5 cm x 4.1 cm was completely removed (Figure 3). The pathological study confirmed the adrenal pheochromocytoma pT3N0R0 (tumor infiltration in surrounding tissue, with no positive lymph nodes and no distance metastases). One year after the surgery, she is clinically stable with continuous endocrinology surveillance.

## Discussion

The increase in antenatal diagnosis of pheochromocytoma is a key factor for the improvement of outcomes in recent years. However, this percentage appears to plateau now at around 70% to 75% of all patients, suggesting that antenatal diagnosis is still missed in approximately one out of three to four patients [5,6].

One possible reason for the delayed diagnosis of pheochromocytoma during pregnancy is the mistaken belief that hypertension in pregnancy is exclusively attributed to gestational hypertension or preeclampsia [6]. Pregnancy-associated hypertension is, undoubtedly, more common than pheochromocytoma, but given the severe consequences of the missing diagnosis during pregnancy, a lower threshold of suspicion is needed. During pregnancy, the clinical presentation closely resembles nonpregnant individuals, with symptoms ranging from mild to severe, including paroxysmal signs and potentially life-threatening cardiovascular complications. Most patients experience a progression of symptoms throughout gestation like in our clinical case [4]. The phenomenon can be attributed to mechanical factors like the expanding uterus, fetal movements, uterine contractions, abdominal palpation, and pharmacological factors [7].

Some clinical differences play a crucial role in the differential diagnosis between pregnancy-related hypertension and hypertension associated with pheochromocytoma, as hypertension is a common symptom [7,8]. In pregnancy, hypertension associated with a pheochromocytoma can occur at any stage, unlike preeclampsia, which is limited to the last 20 weeks of gestation. Paroxysmal symptoms linked to pheochromocytoma are rare in gestational hypertension or preeclampsia. Opposite to gestational hypertension of preeclampsia, pheochromocytoma-related hypertension typically is not associated with edema, proteinuria, or elevated plasma uric acid. Orthostatic hypotension in a pregnant hypertensive woman was pointed out as the most specific symptom indicating a potential pheochromocytoma, especially if no explanation exists [6,7]. Sustained hypertension during pregnancy is linked to negative outcomes, including impaired intrauterine growth, superimposed preeclampsia, and complications in perinatal mortality, which can complicate the process of making a differential diagnosis.

Once there is any clinical suspicion for secondary causes, in cases of uncontrolled hypertension occurring before 20 weeks of pregnancy, for example, clinicians should consider proper biochemical testing [3]. Biochemical tests are the same as those for nonpregnant women. However, there are no clinical studies for assessing the accuracy of specific reference values in the pregnant population [6]. Despite the potential interference of medications, such as methyldopa, in the measurement of urinary catecholamines, our clinical case exhibited a highly significant elevation (a 10-fold elevation above the upper limit of normal values), ruling out the possibility of a false positive [9]. While it is ideal for patients to avoid medication during the diagnostic assessment, the use of antihypertensive medications can be maintained [10]. Despite its widespread availability, speed, and cost-effectiveness, the diagnostic sensitivity of abdominal ultrasonography for detecting pheochromocytoma is limited, especially in the third trimester, where small tumors can be easily overlooked [11]. The main image for diagnosis, and classification of paragangliomas/pheochromocytomas in pregnant women, is MRI [6,12]. Biopsy is not recommended due to the potential risk of triggering a hypertensive crisis [13]. After diagnosis is confirmed, it is important to pursue genetic testing for the identification of specific mutations (such as RET gene; VHL gene; NF1 gene; PGL 1: SDHD; PGL 2: SDHAF2; PGL 3: SDHC; PGL 4: SDHB; PGL 5: SDHA) with increased risk for recurrent or metastatic disease and for earlier diagnosis and treatment for relatives of the proband [6,9]. 

Considering available studies, it is advisable to consider adrenalectomy either before 24 weeks of gestation or during/after delivery [5]. Like in our clinical case, when the diagnosis is at or after 24 weeks, it is recommended to initiate medical treatment with alpha-adrenoceptor blockade, like phenoxybenzamine and doxazosin, to protect the mother from catecholamine excess and defer tumor removal. Delayed surgery after delivery allows to correctly plan surgery after imaging studies with functional MRI. After surgery, a follow-up at two to six weeks is needed to ascertain the surgery's success by measurements of plasma or urine metanephrines [14].

The potential risks associated with labor and vaginal delivery have influenced clinical practices, leading to an inclination toward cesarean delivery. Which seems to offer a more controlled approach, leading to lower maternal morbidity and mortality [15]. Nevertheless, there remains a lack of consensus on the optimal delivery route for pregnant women in these situations [6]. In our case, considering the gestational age, the severity of preeclampsia, and fetal nonreassurance status, the multidisciplinary team decided on cesarean delivery.

## Conclusions

In conclusion, while hypertension due to pheochromocytoma is a rare etiology, pregnancy represents a unique window for its diagnosis. The antenatal diagnosis and subsequent treatment improve the odds of successful maternal and fetal outcomes. Moreover, the typical symptoms of paroxysmal headaches, sweating, and palpitations should prompt obstetricians to consider pheochromocytoma. The prognosis is directly linked to early diagnosis and multidisciplinary management of the pregnant patient.
